# Genetic and Physiological Characteristics of a Novel Marine Propylene-Assimilating *Halieaceae* Bacterium Isolated from Seawater and the Diversity of Its Alkene and Epoxide Metabolism Genes

**DOI:** 10.1264/jsme2.ME18053

**Published:** 2019-01-16

**Authors:** Toshihiro Suzuki, Tomoki Yazawa, Naonori Morishita, Akihiko Maruyama, Hiroyuki Fuse

**Affiliations:** 1 Department of Fermentation Sciences, Faculty of Applied Biosciences, Tokyo University of Agriculture 1–1–1 Sakuragaoka, Setagaya, Tokyo 156–8502 Japan; 2 College of Systems Engineering and Science, Shibaura Institute of Technology Fukasaku 307, Minuma-ku, Saitama city, Saitama 337–8570 Japan; 3 Microbial and Genetic Resources Research Group, Bioproduction Research Institute of Advanced Industrial Science and Technology (AIST) Higashi 1–1–1, Tsukuba, Ibaraki 305–8566 Japan

**Keywords:** propylene assimilation, *Halieaceae* family, marine bacterium, alkene monooxygenase, EaCoMT

## Abstract

The Gram-negative marine propylene-assimilating bacterium, strain PE-TB08W, was isolated from surface seawater. A structural gene analysis using the 16S rRNA gene showed 96, 94, and 95% similarities to *Halioglobus* species, *Haliea* sp. ETY-M, and *Haliea* sp. ETY-NAG, respectively. A phylogenetic tree analysis showed that strain PE-TB08W belonged to the EG19 (*Chromatocurvus*)-*Congregibacter*-*Haliea* cluster within the *Halieaceae* (formerly *Alteromonadaceae*) family. Thus, strain PE-TB08W was characterized as a newly isolated *Halieaceae* bacterium; we suggest that this strain belongs to a new genus. Other bacterial characteristics were investigated and revealed that strain PE-TB08W assimilated propylene, *n*-butane, 1-butene, propanol, and 1-butanol (C3 and C4 gaseous hydrocarbons and primary alcohols), but not various other alcohols, including methane, ethane, ethylene, propane, and *i*-butane. The putative alkene monooxygenase (*amo*) gene in this strain was a soluble methane monooxygenase-type (sMMO) gene that is ubiquitous in alkene-assimilating bacteria for the initial oxidation of alkenes. In addition, two epoxide carboxylase systems containing epoxyalkane, the co-enzyme M transferase (EaCoMT) gene, and the co-enzyme M biosynthesis gene, were found in the upstream region of the sMMO gene cluster. Both of these genes were similar to those in *Xanthobacter autotrophicus* Py2 and were inductively expressed by propylene. These results have a significant impact on the genetic relationship between terrestrial and marine alkene-assimilating bacteria.

Non-methane hydrocarbons (NMHCs) exist naturally in the environment from sources such as vegetation and fuel combustion, and play an important role in chemical production and carbon circulation in the atmosphere. Since NMHCs are highly reactive, they provide a sink for hydroxyl radicals and play a crucial role in the production and destruction of ozone in the troposphere, influencing the photochemical reactions that occur in the atmosphere (11). NMHCs, particularly short-chain alkenes such as ethylene and propylene, are emitted into the environment not only by plants and microorganisms, but also by other natural and artificial sources. Therefore, the microbial degradation of short-chain NMHCs is critically important for global carbon circulation.

Propylene metabolism pathways in microorganisms are characterized by three properties that are distinct from ethylene metabolism pathways: (1) Ethylene is converted to epoxyethane by alkene monooxygenase, which then produces 2-hydroxyethyl-CoM by epoxyalkane:co-enzyme M transferase (EaCoMT); 2-hydroxyethyl-CoM is converted to 2-ketoethyl-CoM by an alcohol dehydrogenase from the short-chain dehydrogenase/reductase (SDR) family (25). In contrast, propylene is converted to the two (*S*)- and (*R*)-epoxypropane enantiomers by alkene monooxygenase, which are then converted to their respective enantiomers of 2-hydroxypropyl-CoM using two types of 2-hydroxypropyl-CoM dehydrogenases (19, 32). The two enantiomers of 2-hydroxypropyl-CoM are converted to 2-ketopropyl-CoM by the *R*- and *S*-forms of 2-hydroxypropyl-CoM dehydrogenase (1, 6, 33). (2) The second difference is that the EaCoMT genes of ethylene-assimilating bacteria are located independently in the upstream region of alkene monooxygenase gene clusters (8), which contain the operon. The EaCoMT genes of propylene-assimilating bacteria are contained in an epoxide carboxylase system with 2-hydroxypropyl-CoM dehydrogenase and 2-ketopropyl-CoM oxidoreductase/carboxylase (6, 17). (3) In ethylene metabolism, 2-ketoethyl-CoM is finally converted to acetyl-CoA by CoM reductase/carboxylase, a bifunctional alcohol/aldehyde dehydrogenase, and CoA transferase. Ethylene-assimilating bacteria possess a CoA-transferase/synthetase cluster near an alkene monooxygenase (25). In propylene metabolism, 2-ketopropyl-CoM is converted to acetoacetate or acetone by 2-ketopropyl-CoM oxidoreductase/carboxylase; therefore, a CoA-transferase/synthetase is not involved in propylene metabolism and the associated genes are not located near the alkene monooxygenase gene cluster. While these findings have been limited to soil bacteria, they show that the lineage of each monooxygenase subunit does not differ between species.

In surface seawater, NMHCs are produced as a by-product of the photochemical transformation of dissolved organic matter (29), but are also produced by micro- and macroalgae, photosynthetic bacteria, and cyanobacteria (5). Therefore, the concentrations of NMHCs in surface seawater are supersaturated, and are at higher levels than in the atmosphere (29), suggesting that carbon circulation in the sea surface, as well as in the terrestrial environment, primarily occurs via the activities of marine microorganisms. We previously isolated an alkene-assimilating bacterium and two ethylene-assimilating bacteria (*Haliea* spp.) from seawater, revealing the initial monooxygenase genes from both strains, which are different from other known alkene-assimilating bacteria (37). Until now, alkene-assimilating marine bacteria have only been reported in our isolates (37), and there is currently a lack of information on marine propylene-assimilating bacteria because no official reports on these organisms or their metabolic capabilities exist. Investigations on the bacterial and genetic characteristics of propylene-assimilating marine bacteria are important for elucidating microbiological dynamics and understanding genetic evolution between terrestrial and marine bacteria.

In the present study, we isolated propylene-assimilating bacteria from seawater and revealed their biological characteristics. Degradation properties were also examined. The complete nucleotide sequences of the putative propylene monooxygenase gene cluster and its related genes involved in epoxide metabolism were elucidated and compared with *amo* and its related genes from other known alkene-assimilating bacteria.

## Materials and Methods

### Bacterial strains and media

Propylene-assimilating bacteria were grown in −C (minus C) medium (100 mg of NH_4_NO_3_, 10 mg of KH_2_PO_4_, 2.5 mg of Fe[III]EDTA, 2.75 mg of vitamin B12, 0.5 mg of biotin, 100 mg of thiamine-HCl, 74.4 mg of Na_2_EDTA, 0.25 mg of CuSO_4_·5H_2_O, 5.75 mg of ZnSO_4_·7H_2_O, 4.55 mg of MnCl_2_·4H_2_O, 0.6 mg of CoCl_2_·6H_2_O, and 0.27 mg of [NH_4_]_6_Mo_7_O_24_·4H_2_O in 1 L of filtered seawater, pH 8.0) with propylene as the sole carbon source. Since propylene is a gas, it was provided by replacing 50% of the air in culture vessels. Plate cultures were made using −C containing 1% gellan gum. Cultures were incubated at 20°C. *Escherichia coli* strains were grown in Luria-Bertani (LB) medium containing 1% polypeptone, 0.5% yeast extract, and 1% NaCl supplemented with ampicillin (100 μg mL^−1^) when necessary.

### Sampling and isolation

Surface seawater was collected from Tokyo Bay, Japan in 120-mL glass vials and cultured in −C liquid medium with propylene. After growth was observed in liquid medium, propylene-assimilating bacteria were purified by serial dilutions with liquid medium and cultured in 96-well plates with propylene. This isolation procedure was repeated five times, at which point the purity of the isolated strain was confirmed by microscopic observations. A transmission electron microscopy (TEM) analysis of purified strains, which were cultured at 22°C for 20 d, was performed by negative staining using JEM-2000EX (Japan Electron Optics Laboratory, Tokyo, Japan). Assessments of DNA G+C contents, cellular fatty acid profiles, and ubiquinones were performed by TechnoSuruga Laboratory (Shizuoka, Japan). Enzyme activity experiments were performed with API ZYM (BioMérieux, Lyon, France) according to the manufacturer’s instructions.

### Identification of polyhydroxybutyrate (PHB)

The extraction of PHB from the isolate PE-TB08W was performed according to the method described by Law and Slepecky (20). To detect PHB, methyl esterification was performed using methanol dehydration and sulfuric acid after chloroform extraction. Putative PHB was extracted by chloroform from PE-TB08W cells grown on propylene. The combined organic phase was dried with NaSO_4_ and concentrated *in vacuo* to obtain crude extracts. The PHB obtained was added to anhydrous methanol and concentrated sulfuric acid (1:1), and the mixture was incubated at 160°C for 3 h. After gently cooling the sample, the same volume of hexane was added. The hexane layer containing crotonate methyl ester was analyzed by TRACE GC ULTRA gas chromatography coupled to a DSQ II mass spectrometer (Thermo Fisher Scientific, Waltham, MA, USA).

### Characteristics of growth

#### Growth temperature

The temperature range for growth was estimated by growing the isolates in liquid −C medium with propylene at 4, 10, 20, 28, 37, and 45°C.

#### Assimilation of gaseous hydrocarbons

Using 120-mL vials, 100 μL of a 100-fold dilution from each pre-culture was inoculated into 25 mL of −C liquid medium and supplied with various gas hydrocarbons or alcohols. The vials were sealed with Teflon-lined rubber septa and then incubated at 20°C for 32 d. Methane, ethane, propane, *n*-butane, *i*-butane, ethylene, propylene, 1-butene, 2-butene, methanol, ethanol, propanol, 1-butanol, 2-butanol, and acetone were added as carbon sources at 50%. Only pentane was added at 0.2%. Except for the propylene culture, growing cells attached to the inner walls of the culture vessels in the liquid medium. Protein concentrations were used as a growth indicator and assessed by the Lowry method (2) using bovine serum albumin as a standard.

#### Effects of salt concentrations

To test the salt dependence of growth, 0, 0.26, 2.6, 13, 26, 52, 79, or 132 g of NaCl was added to 1 L of modified SOW (artificial sea water) medium containing 1.54 g CaCl_2_·2H_2_O, 100 mg KBr, 3 mg KF, 700 mg KCl, 30 mg H_3_BO_3_, 4.09 g K_2_SO_4_, 200 mg KHCO_3_, 17 mg SrCl_2_·6H_2_O, and 11.1 g MgCl_2_·6H_2_O in 1 L of distilled water. The isolates were cultured in these media with propylene as a carbon source at 20°C.

### Genetic manipulations

The isolation of total DNA from PE-TB08W and DNA manipulations for *E. coli* were performed according to the standard protocols by Sambrook *et al*. (30). The 16S rRNA gene coding sequence was amplified using total DNA as a template with the primers 9F (7) and 1510R (41). PCR amplification was performed in a Bio-Rad S1000 Thermal Cycler (Bio-Rad, Hercules, CA, USA) using *Ex Taq* DNA polymerase (Takara, Kyoto, Japan). PCR conditions were as described previously (37). The amplified fragment of the 16S rRNA gene (approximately 1.5 kb) was cloned into the T-vector pMD20 (Takara), and then transformed into *E. coli* DH5α as the host strain. DNA sequencing was conducted by cycle sequencing using the BigDye Terminator v3.1 cycle sequencing kit (Applied Biosystems, CA, USA), and was performed using the ABI PRISM^TM^ 310NT genetic analyzer (Applied Biosystems). Sequencing data were analyzed by GENETYX-MAC software ver. 15 (Genetyx Corporation, Tokyo, Japan). Southern blot hybridization was performed according to the method of Sambrook *et al*. (30). DNA probes were labeled with digoxigenin-11-dUTP (Roche Diagnostics, Mannheim, Germany). Hybridized DNAs were detected by nitrotetrazolium blue chloride (NBT) in combination with 5-bromo-4-chloro-indolyl phosphate (BCIP) solution using an enzyme-linked immunosorbent assay (ELISA).

### Sequences of putative alkene monooxygenase (amo) gene clusters and their flanking regions

All primers used in the present study are listed in Table S1. Degenerate primers were designed from eight other known alkene-assimilating bacteria. The *amoC* gene was obtained by PCR amplification using the forward primer, degenerate-amoC-F3, and the reverse primer, degenerate-amoC-R4. Amplification from part of the *amoA* gene to the *amoC* gene containing the complete *amoB* gene was accomplished using the forward primer, degenerate-amoA-F2, and the reverse primer, PE-amoCR1. Amplification of the *amoD* gene was accomplished using the forward primer, PE-amoD-F1, and the reverse primer, PE-etnD-R5. Finally, amplification from part of the *amoA* gene to the *amoD* gene, containing the complete *amoB* and *amoC* genes, was obtained using the forward primer, PE-seq-F1, and the reverse primer, PED-2R4. The EaCoMT gene was amplified with the forward primer CoM-F1d and the reverse primer CoM-R4d, and the resulting fragments were sequenced. The areas encompassing the upstream region of the *amoA* gene and downstream region of the *amoD* gene, including the flanking region with the partial EaCoMT gene, were obtained using the LA *in vitro* Cloning Kit (Takara Bio, Otsu, Japan). Genomic DNA was digested with the proper restriction enzymes and ligated with the cassette DNAs provided in the kit. PCR was performed according to the manufacturer’s instructions using primers that were designed based on the sequence data obtained in this study in combination with the cassette-specific primers provided in the kit.

### Construction of EaCoMT expression vectors and epoxy alkane conversion

The EaCoMT gene coding sequence was PCR-amplified using total DNA as the template with the primers PE-CoM-Nde and PE-CoM-Bam. The amplified fragment was digested with *Nde*I and *Bam*HI and cloned into pET15b(+) (Novagen, Madison, WI) to obtain pSUPE-15E. pSUPE-15E clones were transformed into *E. coli* BL21(DE3) and cultivated in LB liquid medium containing 100 μg mL^−1^ ampicillin at 28°C for 6 h. They were cultured for a further 16 h after the addition of IPTG (1 mM).

The culture broth of *E. coli* BL21(DE3) harboring pSUPE-15E was harvested by centrifugation at 8,000 rpm and washed once with 50 mM PPB (50 mM potassium dihydrogen phosphate and 50 mM dipotassium hydrogen phosphate, pH 8.0). After centrifugation, the supernatant was discarded, and the cell pellet was resuspended in 50 mM PPB containing 10% (w/v) glycerol at approximately one-tenth of the broth volume. The cell suspension was subjected to ultrasonication (Digital Sonifier; Branson Ultrasonics Corporation, USA). The cell-free extract was collected by centrifugation at 15,000 rpm at 4°C for 15 min. The EaCoMT assay and GC analysis were performed according to the method of Boyd *et al*. (3). Protein concentrations were assessed according to Bradford (4) using bovine serum albumin as a standard.

### RNA manipulations

PE-TB08W isolates were grown for one week in −C liquid medium with 50% propylene and *n*-butane in the gas phase and 0.3% 1-butanol in the medium; cells were cultivated at 20°C for ten d. Total RNA was extracted using the RNeasy Mini Kit (Qiagen K. K., Tokyo, Japan) according to the manufacturer’s instructions. Trace amounts of DNA were removed with RQ1 RNase-Free DNase (Promega, Madison, WI, USA). A reverse transcription-polymerase chain reaction (RT-PCR) was performed with OneStep RT-PCR Kit Ver. 2 (Takara, Tokyo, Japan). RT-PCR without the reverse transcription step was used as a control and was performed using *Ex* Taq DNA polymerase (Takara). Reverse transcription and cDNA synthesis were performed at 50°C for 30 min and 94°C for 2 min, followed by 25 cycles of 96°C for 30 s, 58°C for 30 s, and 72°C for 1 min.

### Phylogenetic analysis

Genetic analyses were conducted using the 16S rRNA gene of strain PE-TB08W, which has been deposited in GenBank. Homology searches were conducted using the BLAST program (http://www.ncbi.nlm.nih.gov/BLAST/). Sequences were aligned with the CLUSTALW ver. 1.83 program. Phylogenetic trees were constructed with TreeViewX software using the neighbor-joining method. A bootstrap analysis with 100 trial replications was performed to assess the reliability of clustering patterns.

### Genbank accession

The gene accession number assigned to the 16S rRNA gene of the strain PE-TB08W is AB728559. The gene accession number for the ~20-kb DNA sequence containing the putative *amo* gene cluster and adjacent alkene metabolism-related genes is AB728560.

## Results and Discussion

### Isolation and characterization of the propylene-assimilating bacterium, strain PE-TB08W

**Fig. 1:**
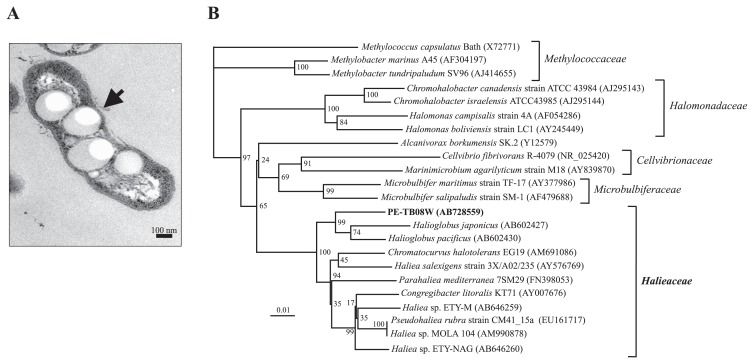
(**A**) Transmission electron microscopy analysis of strain PE-TB08W. The arrow indicates accumulated PHB. (**B**) Phylogenetic tree of 16S rRNA genes constructed using a neighbor-joining algorithm. *Methylococcus capsulatus* served as the outgroup. The numbers on the right are GenBank accession numbers, and the scale bar indicates 0.01 substitutions per 100 base positions. Bootstrap values from 100 trials are listed at the tree nodes. Bacterial families were examined by “List of Prokaryotic names with Standing in Nomenclature” (LPSN; http://www.bacterio.net).

We isolated one propylene-assimilating bacterium, strain PE-TB08W, from the surface seawater of Tokyo Bay, Japan using an enrichment culturing with 50% propylene as the carbon source. This strain was Gram-negative and formed cream-colored colonies on the −C-gellan gum plate with propylene, but rarely grew on Marine Agar 2216 (MA) plates. Using the TEM analysis, we observed the cell type and dimensions for this isolate as a non-flagellated, short rod that was 0.3–0.4 μm in width and 1.2–1.4 μm in length (Fig. 1A). The TEM analysis also showed the low electron density structure of the cell membrane and revealed the accumulation of PHB. Notably, *trans*- and *cis*-crotonic acids resulted in the methyl-esterification of PHB (27) and were detected by the GC/MS analysis (data not shown). The optimum growth temperature was 20°C, and optimum salinity range was 26.4 g L^−1^, similar to *Haliea* sp. ETY-NAG (37). Cellular fatty acids were composed of four primary fatty acids: C18:1ω7c, C10:0 3OH, C16:1ω7c or C15:0 iso 2OH, and C16:0, similar to *Haliea* sp. ETY-M (37). The quinone was ubiquinone Q-8, as observed in other *Haliea* species. The DNA G+C content was 55.1 mol%, which was lower than 57.8, 63.0, and 55.8–65.2 mol% observed in *Congregibacter litoralis* KT71 (34), *Chromatocurvus halotolerans* EG19 (10, 35), and other *Haliea* species (21, 23, 37–39), respectively. The *Halieaceae* family was re-classified from the *Alteromonadaceae* family in 2015 and belongs to the order *Cellvibrionales* (36). *Halieaceae* family bacteria are gammaproteobacteria, Gram-negative, aerobic, and marine, and have often been isolated from coastal, open, and deep-sea waters (15). Other *Halieaceae* family bacteria also grow in the presence of NaCl under aerobic conditions and produce pigments; however, the reported colors differ. Park *et al*. recently reported that two *Halioglobus* species, *H. japonicus* and *H. pacificus* (Gram-negative gammaproteobacteria), were isolated from the seawater of the Northwestern Pacific Ocean (28). Their salinity range, growth temperature, and quinone systems were very similar to our PE-TB08W isolate. The characteristics of PE-TB08W were compared with other bacteria from the *Halieaceae* family in Table 1.

A 16S rRNA gene sequence analysis was performed to examine the phylogeny of this strain. The sequence analysis showed 96, 94, and 95% similarities to *Halioglobus* species, *Haliea* sp. ETY-M, and *Haliea* sp. ETY-NAG, respectively. The phylogenetic tree showed that strain PE-TB08W belonged to the EG19 (*Chromatocurvus*)-*Congregibacter*-*Haliea* cluster (10) in the *Halieaceae* family (36), which are members of the OM60/NOR5 clade (35); however, PE-TB08W formed a new branch near the *Halioglobus* species (Fig. 1B). Based on these results, PE-TB08W was assumed to be a new species or even a new genus within the *Halieaceae* family of bacteria.

### PE-TB08W assimilated C3 and C4 gaseous hydrocarbons and primary alcohols

**Fig. 2:**
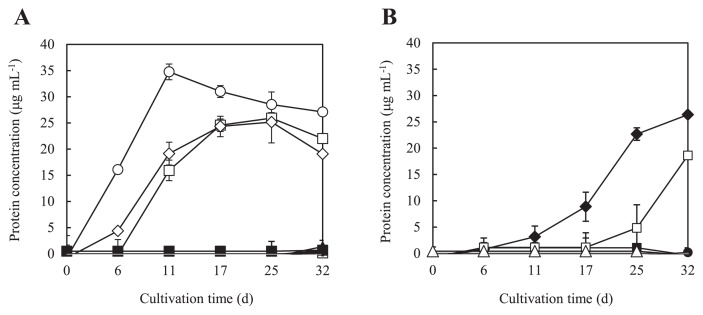
Growth curves of strain PE-TB08W using various growth substrates. The longitudinal axis indicates protein concentrations (μg mL^−1^) using the Lowry method. The horizontal axis indicates cultivation times (d). Results are plotted as the mean values obtained for at least two experiments. Error bars represent the range of individual points. (**A**) Assimilation of gaseous hydrocarbons: (●), Methane; (■), Ethane; (▲), Propane; (◆), Ethylene; (○), Propylene; (□), *n*-butane; (△), *i*-butane; (⋄), 1-butene; (▼), *cis*-2-butene; (▽), *trans*-2-butene; (×), control (no gaseous hydrocarbon substrate added). (**B**) Assimilation of alcohols: (●), Methanol; (■), Ethanol; (▲), Acetone; (◆), 1-propanol; (○), 2-propanol; (□), 1-butanol; (△), 2-butanol; (⋄), Pentane; (×), control (not alcohol added as a substrate).

The assimilation of gaseous hydrocarbons and alcohols was examined. PE-TB08W assimilated *n*-butane and 1-butene as gaseous hydrocarbons, as well as propylene (Fig. 2A), but not other alkenes. The most well-known propylene-assimilating bacteria are *Rhodococcus rhodochrous* B-276 and *Xanthobacter autotrophicus* Py2. *R. rhodochrous* B-276 has been shown to grow on ethylene, propylene, propane, 1-butene, and butane, but not on methane or ethane (12). On the other hand, *X. autotrophicus* Py2 has been shown to utilize ethylene and propene, but not alkanes, such as ethane, propane, or butane (40). Propylene-utilizing *Mycobacterium* strains, such as *Mycobacterium* sp. M156, were shown to grow on propylene and 1-butene, but not ethylene or saturated alkanes (42). It is noteworthy that gaseous hydrocarbon assimilation patterns differed among alkene-assimilating bacteria. The genetic characterization of these bacteria differs from species to species. The alkene monooxygenase gene components of the CMNR group, including *R. rhodochrous* B-276, comprise four open reading frames, while the alkene monooxygenase gene components of *X. autotrophicus* Py2 comprise six open reading frames (43) (see below). Some strains of bacteria contain several types of monooxygenases, such as *Mycobacterium chubuense* NBB4, which is able to grow on alkenes (ethene, propene, butene) and alkanes (ethane, propane, butane, pentane, hexane, heptane, octane, and hexadecane) (9).

Regarding the assimilation of alcohols, PE-TB08W grew well on 1-propanol and 1-butanol, but not on methanol, ethanol, acetone, 2-propanol, 2-butanol, or pentane (Fig. 2B). *X. autotrophicus* Py2 was able to utilize various alcohols, including 1-propanol and 1-butanol (40), while *R. rhodochrous* B-276 grew on 1,2-propanediol (12). Within the former *Alteromonadaceae* family, *Microbulbifer* strains were not able to utilize all alcohols, while *Marinimicrobium* strains were able to utilize methanol, ethanol, 1-propanol, 2-propanol, and 1-butanol (14). *Haliea* spp. ETY-M and ETY-NAG were only able to grow on ethanol (37). With the exception of strains ETY-M and ETY-NAG, *H. salexigens* (38), *H. mediterranea* (23), and *Pseudohaliea rubra* (formerly *Haliea rubra*) (35, 39), all other known members of the *Haliea* genus were unable to assimilate ethylene and propylene (37). However, it is notable that the assimilation of gaseous hydrocarbons has not been reported in the *Halieaceae* family, except for *Haliea* sp. ETY-M and ETY-NAG. These results suggest that differences in gaseous hydrocarbons and alcohol-assimilation patterns are related to those in the genes and gene organization in each microorganism.

### Nucleotide sequence of a 20-kb region containing genes related to alkene assimilation

**Fig. 3:**
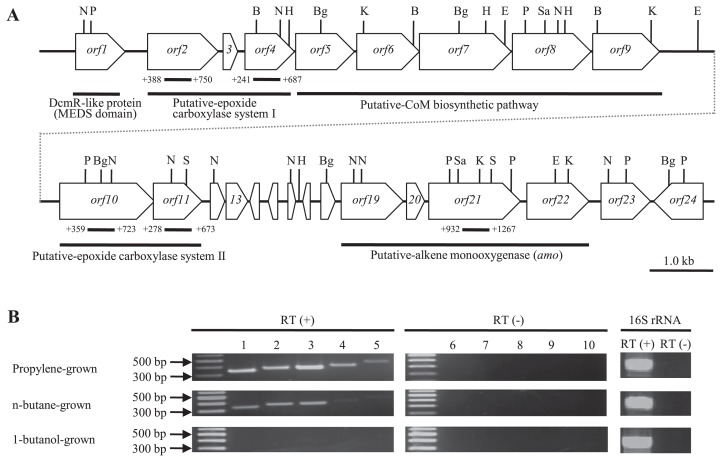
Gene organization of approximately 20 kb containing the *amo* gene cluster and its flanking region in PE-TB08W (**A**) N, *Nde*I; P, *Pst*I; B, *Bam*HI; H, *Hind*III; Bg, *Bgl*II; E, *Eco*RI; Sa, *Sac*I; S, *Sal*I; K, *Kpn*I. Black lines indicate the region amplified by RT-PCR. Numbers on the side indicate positions from the start codon of each gene. (**B**) RT-PCR analysis. Transcriptional analysis of the *amoC*, EaCoMT, 2-KPCC, *S*-HPCDH, and *R*-HPCDH genes. The upper panel shows the transcription of each mRNA in propylene-grown cells. The middle panel shows the transcription of each mRNA in *n*-butane-grown cells. The lower panel shows the transcription of each mRNA in 1-butanol-grown cells. Lane 1, *amo*C; lane 2, EaCoMT gene; lane 3, 2-KPCC gene; lane 4, *S*-HPCDH gene; lane 5, *R*-HPCDH gene; lanes 6–10, non-RT corresponding to lanes 1–5, respectively.

**Fig. 4:**
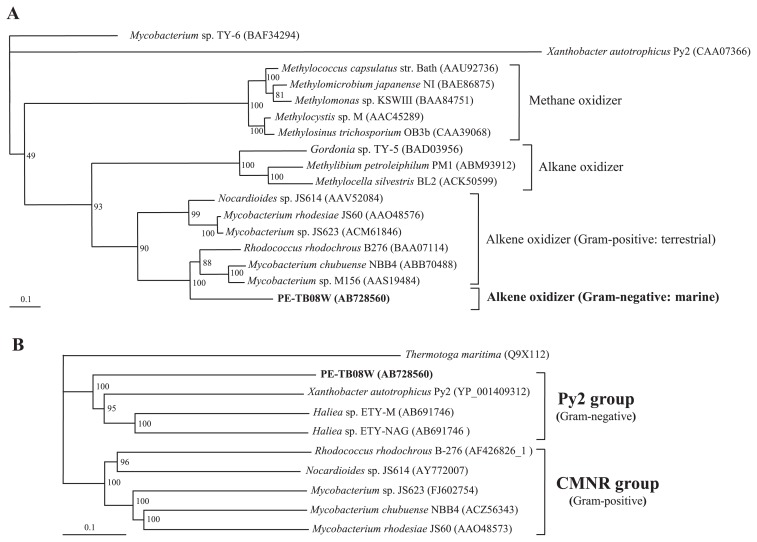
Phylogenetic tree of deduced amino acid sequences of alkene monooxygenase α-subunits (**A**) and EaCoMT genes (**B**) using the neighbor-joining algorithm. Numbers on the right are accession numbers in the database. The scale bar indicates 0.1 substitutions per 100 base positions. Bootstrap values from 100 trials are listed at the tree nodes. In the phylogenetic tree of EaCoMT, *Thermotoga maritima* (Q9X112) served as an outgroup.

Nearly all alkene-assimilating bacteria possess an initial monooxygenase that is a soluble methane monooxygenase-like (sMMO-like) gene, except for the marine *Haliea* spp. ETY-M and ETY-NAG (37), which possess a *pmo*-like gene. To detect this gene in PE-TB08W, we performed Southern blot hybridization using the *pmoA*-like gene from ETY-M and ETY-NAG as a probe and PCR with the universal primer for *pmoA*, which was designed from the *pmoA*-like genes of ETY-M and ETY-NAG. However, no *pmo*-like genes were detected in PE-TB08W total DNA (data not shown). In order to elucidate the sMMO-like gene sequences that are possessed extensively by alkene-assimilating bacteria, degenerate primers were designed from *amoC*, which encodes the alpha subunit of other known alkene-assimilating bacteria. We were ultimately able to sequence ~20 kb of the flanking region of the *amoC*-like gene by genome walking, resulting in a complete *amo*-like gene cluster and its various functional genes. These regions contained 24 open reading frames, as shown in Fig. 3A. The 20-kb region contained a putative alkene monooxygenase (*amo*) gene cluster (ORF19–ORF22), which is similar to other known alkene-assimilating bacteria, such as the CMNR group. The phylogenetic tree analysis showed that the deduced amino acid sequence of *amoC* formed a new branch near the monooxygenases of alkene-assimilating terrestrial bacteria (Fig. 4A). In addition to finding this putative *amo* gene cluster, two putative epoxide carboxylase systems were located around the clusters and were shown to contain epoxyalkane:co-enzyme M transferase (EaCoMT) (ORF2–ORF4 and ORF10–ORF11), a co-enzyme M biosynthetic gene cluster (ORF5–ORF9), and other genes (ORF1, ORF12–18, and ORF23–ORF24). Each deduced amino acid in these genes, except for in the putative *amo* gene cluster, was very similar to its counterpart in *X. autotrophicus* Py2, while the order in the putative *amo* gene cluster was the same as in other known alkene-assimilating bacteria. Predicted gene products and database similarities are shown in Table S2.

### A single copy of the EaCoMT gene exists in the PE-TB08W genome

EaCoMT genes have been found in many alkene-assimilating bacteria, such as the CMNR group and *X. autotrophicus* Py2 (6, 8, 24, 43). When we searched for homologous EaCoMT genes using a Southern blot hybridization analysis (data not shown), a single copy of a putative EaCoMT gene (ORF2) existed in the epoxide carboxylase system (ORF2–ORF4) of PE-TB08W. The deduced amino acid sequence showed 66, 63, and 62% similarities to the EaCoMTs of *X. autotrophicus* Py2, *Haliea* sp. ETY-M, and *Haliea* sp. ETY-NAG, respectively. The phylogenetic tree showed that the putative EaCoMT gene of PE-TB08W was the most closely related to the 2-hydroxypropyl-CoM lyase of *X. autotrophicus* strain Py2 (Fig. 4B), and together with *Haliea* spp. ETY-M and ETY-NAG constituted the EaCoMT Py2 group.

The EaCoMT gene plays a critical role in alkene assimilation (22) because EaCoMT adds co-enzyme M to epoxyalkane during alkene degradation. To examine the function of EaCoMT, we constructed the expression plasmid pSUPE-15E and examined the conversion of various epoxy alkane compounds (epoxypropane, epoxyethane, and epoxybutane) using the recombinant EaCoMT. Table 2 shows the residual epoxyalkane from the cell-free extract conversion. While a crude extract from *E. coli* harboring pET15b(+) did not decrease the amount of any epoxyalkane, the recombinant EaCoMT showed conversion activity against some epoxyalkanes. Epoxypropane, a mixture of chiral epoxides ((*R*)- and (*S*)-epoxypropane), decreased by 70% from that of the control following the addition of recombinant EaCoMT, while (*R*)- and (*S*)-epoxypropane were decreased by 30.2 and 61.0%, respectively, from that of the control. Similar to epoxypropane, epoxyethane was markedly decreased (15.8%) by the addition of the recombinant EaCoMT.

In contrast to the above epoxy compounds, 1,2-epoxybutane; *cis*-2,3-epoxybutane; and *trans*-2,3-epoxybutane did not show a marked decrease. In the cases of ethylene-assimilating bacteria, their EaCoMT were able to decrease epoxyethane, but not epoxypropane (Suzuki, submitted). These results suggest that the EaCoMT of PE-TB08W exhibits epoxy compound conversion activity, which plays a key role in propylene metabolism.

### Transcriptional analysis and localization of genes related to alkene metabolism

To examine whether the putative *amo* genes and epoxide carboxylase systems of strain PE-TB08W were expressed inductively or constitutively by propylene and if they functioned in the oxidation of propylene, we performed a transcriptional analysis using reverse transcription-PCR. Growth substrates were used for propylene, *n*-butane, and 1-butanol. Total mRNAs were isolated from propylene-grown and *n*-butane-grown cells at ten d and were isolated from 1-butanol-grown cells at 25 d according to the growth of each (Fig. 2A and B). The indicators for each gene cluster used were the *amoC*, EaCoMT, 2-ketopropyl-CoM oxidoreductase/carboxylase (2-KPCC), (*R*)-2-hydroxypropyl-CoM dehydrogenase (*R*-HPCDH), and (*S*)-2-hydroxypropyl-CoM dehydrogenase (*S*-HPCDH) genes. When propylene-grown cells were used, mRNAs were detected for all genes. These results suggest that all the putative *amo* genes and epoxide carboxylase systems were inductively expressed in the presence of propylene, indicating that they function in the oxidation of propylene. In the putative *amo* gene cluster, a putative sigma54-like promoter sequence (24) (**GG**CCCCCAGGTCG A**GC**AGC; boldface indicates −24 and −12 elements) existed in a 193-bp region upstream of the *amoA* start codon, while a putative stem-loop sequence (**ATGATGAG**CCTCCTCGGA A**CACAGCAT**; boldface indicates stem sequences) existed in the region downstream of *amoD*. A putative Shine-Dalgarno sequence was identified in the upstream region of each putative *amo* gene. This result also suggests that putative *amo* genes constitute an operon structure. In addition, putative *amo* genes may be expressed separately from epoxide carboxylase systems via some regulatory systems.

Very thin bands were detected in *S*-HPCDH and *R*-HPCDH (Fig. 3B; lanes 4 and 5) in *n*-butane-grown cells; however, it is unlikely that these genes are involved in *n*-butane assimilation because they act on their respective enantiomers of 2-hydroxypropyl-CoM in propylene metabolism (19, 32). *S*-HPCDH and *R*-HPCDH are a part of putative epoxide carboxylase system I and putative epoxide carboxylase system II, respectively, and are present in the region downstream with EaCoMT and 2-KPCC (Fig. 3A). Since EaCoMT and 2-KPCC were transcribed in *n*-butane-grown cells, *S*-HPCDH and *R*-HPCDH appear to constitute an operon with EaCoMT and 2-KPCC, respectively, because promoter-like sequences were not found in their upstream regions. Kotani *et al*. reported that the propane hydroxylase genes in *Gordonia* sp. TY-5 and *Mycobacterium* sp. TY-6 comprised the following four components: (1) a hydroxylase large subunit, (2) a reductase, (3) a hydroxylase small subunit, and (4) a coupling protein (18). Thus, the putative *amo* genes in PE-TB08W may also function in butane assimilation. PE-TB08W was positive for *n*-butane assimilation, whereas *X. autotrophicus* Py2 did not have the ability to assimilate alkanes. A recent study reported that *Mycobacterium chubuense* NBB4 possesses a *pmo*-like gene homolog (*pHMO*), and the heterologous expression of *pHMO* led to a decrease in ethane, propane, and butane (9). *Nocardioides* sp. CF8 also possesses a *pmo*-like gene homolog (*pBMO*) in addition to alkane hydroxylase genes (31). However, no *pmo*-like genes were detected in PE-TB08W total DNA. In contrast, no mRNAs were detected for any of the indicator genes in cells grown on 1-butanol (Fig. 3B) with any of the growth substrates used, suggesting that neither putative epoxide carboxylase systems nor putative *amo* genes were involved in 1-butanol metabolism.

To examine whether the putative *amo* genes and epoxide carboxylase systems are essential for propylene assimilation, we attempted to express the putative *amo* genes and disrupt the putative amo genes and epoxide carboxylase systems; however, we were unsuccessful. The putative *amo* gene was expressed in the presence of propylene as the sole carbon and energy source in RT-PCR. In addition, putative *amo* genes constitute an operon and are present near the regions of epoxide carboxylase systems and the putative CoM biosynthetic pathway. These results strongly suggest that the putative *amo* gene functions as an alkene monooxygenase of PE-TB08W.

### Positional relationship between the putative amo gene cluster and EaCoMT gene

**Fig. 5:**
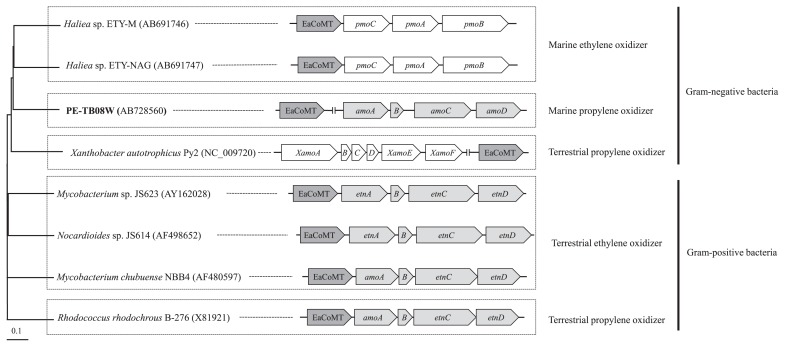
Relationships between 16S rRNA, alkene monooxygenase, and EaCoMT genes from various alkene-assimilating bacteria. Phylogenetic tree of 16S rRNA genes constructed using a neighbor-joining dendrogram. The scale bar indicates 0.1 substitutions per 100 base positions. Numbers in parentheses on the right of the strain name are accession numbers in the database. Components of the EaCoMT and alkene monooxygenase gene cluster are represented by different colors: EaCoMT gene, gray; soluble methane monooxygenase-type (*amo* or *etn*) genes, light gray; *xamo* and particulate methane monooxygenase (*pmo*)-type genes, white.

Observations of sequence data, assimilation data, and transcriptional data from the PE-TB08W isolate gradually clarified the order of the EaCoMT/*amo* genes in the relationship between terrestrial and marine alkene-assimilating bacteria. The location of the EaCoMT gene differed among ethylene-and propylene-assimilating bacteria (Fig. 5). The ethylene-assimilating terrestrial bacteria, *Nocardioides* sp. JS614 and *Mycobacterium* strain JS60, possess EaCoMT genes immediately upstream of their alkene monooxygenase gene clusters (8, 16). In contrast, *XecA* of *X. autotrophicus* strain Py2 is part of the epoxide carboxylase system that does not form an operon. Although the EaCoMT gene of *Mycobacterium* strain JS60 (8) is co-transcribed with the *amo* gene cluster, the EaCoMT gene of marine ethylene-assimilating *Haliea* sp. ETY-M is co-transcribed with the *emo* gene cluster (Suzuki, submitted). These findings explicitly suggest a difference in genetic diversity between terrestrial and marine gaseous hydrocarbon-assimilating bacteria.

It is particularly noteworthy that EaCoMT and other gene clusters from PE-TB08W belong to the Py2 group, whereas the putative *amo* genes were more closely related to the CMNR group. Furthermore, the *amo* gene cluster and EaCoMT phylogeny did not reflect 16s rRNA gene phylogeny and the location of the EaCoMT gene differed from species to species (Fig. 5). There were seven truncated genes (*orf12-18*) reading in the opposite direction between the putative epoxide carboxylase system and *amo* gene cluster of PE-TB08W (Fig. 3A). Although each of the *amo* genes in PE-TB08W and their order were more similar to those in the CMNR group than in *X. autotrophicus* Py2, the epoxide carboxylase system is more closely related to the latter. Based on these results, we speculated that the gene arrangements in strain PE-TB08W were constituted with non-homologous recombination or horizontal gene transfer to enable efficient propylene assimilation in the marine environment; genetic transformation within a host genome occurs rapidly in this environment (26). Our supposition is also supported by the existence of the transposase/integrase gene and truncated genes that were similar to those in *Pseudoalteromonas haloplanktis* and the genus *Shewanella*, both of which are marine microorganisms (13).

Alkene-assimilating marine bacteria belonging to the *Halieaceae* family have only been isolated in our laboratory. Among this family of bacteria, the assimilation of other gaseous hydrocarbons, related genes, and similar genetic characterizations could be common to each microorganism. Future studies to examine further gaseous hydrocarbon assimilation and additional common genes within members of the *Halieaceae* family will be important for elucidating microbial dynamics together with genetic relationships between terrestrial and marine bacteria groups. The isolation of many other gaseous hydrocarbon-assimilating marine bacteria is greatly desired.

## Supplementary Information



## Figures and Tables

**Table 1:** Comparison of PE-TB08W and other *Halieaceae* family bacteria.

Characteristics	Strains					

	PE-TB08W	*Halioglobus pacificus* S1-72^T^	*Haliea salexigens* 3X/A02/235^T^	*Haliea* sp. ETY-M	*Chromocurvus halotolerans* EG19^T^	*Congregibacter litoralis* KT71^T^
Isolation source	Surface seawater	Surface seawater	Surface seawater	Surface seawater	Spring run-off stream	Seawater (depth of 8 m)
Cell morphology	Short Rods	Coccus	Straight rods	Short Rods	Pleomorphic	Pleomorphic
Cell dimensions (mm)	0.4–0.45×1.2–1.3	0.3×0.5	0.3–0.7×1.3–1.9	0.4–0.45×1.2–1.3	0.7×1.5–3.0	0.5–4.5×0.4–0.7
Colony color (agar medium)	Cream (−C)	N.A.	Cream (MA)	Purple (5VM)	Pale pinkish-purple (Medium A)	Cream (MA) Pale yellow to orange-red (Under certain conditions)
Growth of nutrient agar (MA)	−	+	+	−	N.A.	+
Flagella	−	−	+ (1 polar)	−	N.A.	+ (1–2 polar)
DNA G+C content (mol%)	55.1	59.4	61.4	65.2	63.0	57.8
Optimum growth temperature (°C)	20	20–25	25–30	30	37	28
Optimum salt concentration (g L^−1^)	26.4	20	40	13.2	40	20
PHB accumulation	+	N.A.	N.A.	−	N.A.	N.A.
Quinone	Ubiquinone Q-8	Ubiquinone Q-8	Ubiquinone Q-8	Ubiquinone Q-8	Ubiquinone Q-8	Ubiquinone Q-8
Major fatty acid compositions	C18:1ω7c, C16:1ω7c or C15:0 iso 2OH, C16:0, C14:0, C10:0 3OH	C18:1ω7c, C16:1ω7c, C17:1ω8c, C11:0	C18:1ω7c, C17:1ω8c, C16:1ω7c, C17:0	C18:1ω7c, C16:1ω7c or C15:0 iso 2OH, C16:0, C14:0, C10:0 3OH	N.A.	C18:1ω7c, C16:1ω7c, C16:0, C10:0 3OH
Gas hydrocarbon utilization of						
Ethylene	−	N.A.	N.A.	+	N.A.	N.A.
Propylene	+	N.A.	N.A.	−	N.A.	N.A.
1-butene	+	N.A.	N.A.	−	N.A.	N.A.
2-butene	−	N.A.	N.A.	−	N.A.	N.A.
Methane	−	N.A.	N.A.	−	N.A.	N.A.
Ethane	−	N.A.	N.A.	−	N.A.	N.A.
Propane	−	N.A.	N.A.	−	N.A.	N.A.
*n*-butane	+	N.A.	N.A.	−	N.A.	N.A.
*i*-butane	−	N.A.	N.A.	−	N.A.	N.A.
Alcohol utilization of						
Methanol	−	N.A.	N.A.	−	—	—
Ethanol	−	N.A.	N.A.	+	—	—
1-propanol	+	N.A.	N.A.	(+)	N.A.	N.A.
2-propanol	−	N.A.	N.A.	−	N.A.	N.A.
1-butanol	+	N.A.	N.A.	−	N.A.	N.A.
2-butanol	−	N.A.	N.A.	−	N.A.	N.A.
Pentane	−	N.A.	N.A.	−	N.A.	N.A.

MA, marine agar 2216; +, positive; −, negative; N.A., data not available.

**Table 2:** Oxidation of various epoxyalkanes by recombinant EaCoMT in PE-TB08W

Substrates	Relative residual substrates (%)	

	Control	Recombinant EaCoMT
Epoxypropane	89.4±1.11	62.6±1.39
(*R*)-(+)-epoxypropane	92.8±0.12	28.0±0.73
(*S*)-(+)-epoxypropane	90.2±0.54	55.1±0.41
Epoxyethane	96.2±0.22	15.2±0.21
1,2-epoxybutane	100.4±0.20	99.6±0.20
*cis*-2,3-epoxybutane	91.9±0.25	78.7±0.10
*trans*-2,3-epoxybutane	105.9±0.07	96.9±0.24

Epoxypropane contains (*R*)-(+)-epoxypropane and (*S*)-(−)-epoxypropane. Since epoxyethane is a liquid, 1.2 mL (5 μmol) of epoxyethane was injected into a vial. One-hundred-milliliter samples were injected into a gas chromatographic system. Data are mean values in duplicate represented as means±standard deviations.
