# Supplementary material for: Genetic and Physiological Characteristics of a Novel Marine Propylene-Assimilating Halieaceae Bacterium Isolated from Seawater and the Diversity of Its Alkene and Epoxide Metabolism Genes
Source: Microbes Environ. 2019 Jan 16;34(1):33–42. doi: 10.1264/jsme2.ME18053 (PMC6440738; doi:10.1264/jsme2.ME18053)
Supplement: Supplementary file 1 [file 34_33_s1.pdf]

Table S1. Oligonucleotides used in this study.

Primer name	Sequence (5'-3')	References
PE-RT-amoC-F	TATTGGGTAAAGCCGTGGAAGAGTT	This study
PE-RT-amoC-R	ATGTTGGATACTGCTTCCCCTTGAT	This study
PE-RT-EaCoMT-F	ACGACGAAGCCAATTAAAATCCAGT	This study
PE-RT-EaCoMT-R	ATAGGACTCAACTTTCCGCGAACTC	This study
PE-RT-2KPCC-F	CAGCAGAACTGATGTTGAATCGAGA	This study
PE-RT-2KPCC-R	AACTGCAGTATCACCAGGCTCGTAG	This study
PE-RT-RHPCDH-F	ATCAGTCCGCTATGGAATTACACATT	This study
PE-RT-RHPCDH-R	CTGTGCCCGCTACATCGTCGACACT	This study
PE-RT-SHPCDH-F	GTCAGCAAAGCACTTTCGAATAG	This study
PE-RT-SHPCDH-R	TATTTCAGAAACGCTGGCAAATCTCC	This study
9F	GAGTTTGATCCTGGCTCAG	Brosius 1981 (7)
1510R	GGTTACCTTGTTACGACTT	Weisburg 1991 (41)
degenerate-amoC-F3	TGGGARGARTGGGTIGTIGAYGAYTTYGTI	This study
degenerate-amoC-R4	ICKDATRTCRTCDATIGTCCA	This study
degenerate-amoA-F2	TGGCCIBTIMRITTYGAYRAYGGI	This study
PE-amoCR1	TGGATACTGCTTCCCCTTGATGTCTATTAATCGAAATTCATTAATG	This study
PE-amoD-F1	AARTAYGGITGYAARCAYGGIGGITGY	This study
PE-etnD-R5	IGGIGCIADICCIGAICCICCIGCDAT	This study
PE-seq-F1	CTTCAAACGACTGGGCTGAGATTGTTCTAG	This study
PED-2R4	CAAGGATTTACGCGTAGGAGATAAGTTAGTAGTACATGGC	This study
PE-CoM-Nde	CTATTAATCATATGTCACAAGCTACCCCGCTCATCC	This study
PE-CoM-Bam	TAAGATCTGGATCCTTAAGCTAGCTCCGCTCGAACAAT	This study

Table S2 Putative gene products and database similarities of PE-TB08W

ORF No.	Predictive gene products	AA	Protein name and Organism (accession number)	Identity (%)
ORF1	DNA binding protein containing MEthanogen/methylotroph, DcmR Sensory domain (MEDS)	243	DNA binding domain-containing protein, Xaut_4864: *Xanthobacter autotrophicus* Py2 (YP_001409311)	31
			DNA-binding protein, excisionase family: *Methylomicrobium album* BG8 (ZP_09865456)	37
ORF2	Epoxyalkane:coenzyme M transferase (EaCoMT)	369	2-hydroxypropyl-CoM lyase, Xaut_4865: *Xanthobacter autotrophicus* Py2 (YP_001409312)	66
			Epoxyalkane:coenzyme M transferase: *Haliea* sp. ETY-M	63
			Epoxyalkane:coenzyme M transferase: *Haliea* sp. ETY-NAG	62
			Epoxyalkane:coenzyme M transferase: *Rhodococcus rhodochrous* B-276 (AF426826)	46
			Epoxyalkane:coenzyme M transferase: *Mycobacterium chubuense* NBB4 (ACZ56343)	45
ORF3	Hypothetical protein	64	Fumarate lyase, Xaut_4866: *Xanthobacter autotrophicus* Py2 (YP_001409313)	43
ORF4	(*S*)-2-Hydroxypropyl-CoM dehydrogenase (*S*-HPCDH)	259	*S*-HPCDH, Xaut_4869: *Xanthobacter autotrophicus* Py2 (YP_001409316)	49
			S-HPCDH, Xaut_5050: *Xanthobacter autotrophicus* Py2 (YP_001409484)	49
			S-HPCDH, Xaut_5073: *Xanthobacter autotrophicus* Py2 (YP_001409507)	48
ORF5	Hypothetical protein	312	Hypothetical protein, Xaut_5051: *Xanthobacter autotrophicus* Py2 (YP_001409485)	59
			Hypothetical protein, Xaut_5070: *Xanthobacter autotrophicus* Py2 (YP_001409504)	57
			Hypothetical protein: *Nocardioides* sp. JS614 (YP_919264)	44
ORF6	Phosphosulfolacetate synthase (ComA)	294	Phosphosulfolactate synthase, Xaut_4872: *Xanthobacter autotrophicus* Py2 (YP_001409317)	54
			Phosphosulfolactate synthase, Xaut_5052: *Xanthobacter autotrophicus* Py2 (YP_001409486)	54
			Phosphosulfolactate synthase, Xaut_5069: *Xanthobacter autotrophicus* Py2 (YP_001409503)	54
ORF7	Fumarate lyase	510	Argininosuccinate lyase, Xaut_4873: *Xanthobacter autotrophicus* Py2 (YP_001409318)	43
			Fumarate lyase, Xaut_5053: *Xanthobacter autotrophicus* Py2 (YP_001409487)	44
			Argininosuccinate lyase: *Nocardioides* sp. JS614 (YP_919267.1)	35
ORF8	Fumarate lyase	451	Fumarate lyase, Xaut_4874: *Xanthobacter autotrophicus* Py2 (YP_001409319)	47
			Fumarate lyase, Xaut_5054: *Xanthobacter autotrophicus* Py2 (YP_001409488)	45
			Fumarate lyase: *Nocardioides* sp. JS614 (YP_919284.1)	43
ORF9	1-Aminocyclopropane-1-carboxylate deaminase (ACC)	337	ACC deaminase, Xaut_4875: *Xanthobacter autotrophicus* Py2 (YP_001409320)	49
			ACC deaminase , Xaut_5055: *Xanthobacter autotrophicus* Py2 (YP_001409489)	49
			ACC deaminase: *Mycobacterium rhodesiae* NBB3 (YP_005000702)	41
ORF10	2-Ketopropyl-CoM oxidoreductase/carboxylase (2-KPCC)	525	2-KPCC, Xaut_4867: *Xanthobacter autotrophicus* Py2 (YP_001409314)	67
			2-KPCC, Xaut_5048: *Xanthobacter autotrophicus* Py2 (YP_001409482)	67
			Putative 2-ketopropyl-CoM carboxylase: *Mycobacterium chubuense* NBB4 (ACZ56354)	53

ORF11	(*R*)-2-Hydroxypropyl-CoM dehydrogenase (*R*-HPCDH)	250	*R*-HPCDH, Xaut_4868: *Xanthobacter autotrophicus* Py2 (YP_001409483)<br>*R*-HPCDH, Xaut_5049: *Xanthobacter autotrophicus* Py2 (YP_001409508)<br>Short-chain dehydrogenase/reductase SDR: *Nocardioides* sp. JS614 (YP_919273)	64<br>64<br>45
ORF12	Hypothetical protein	62	No similarity	–
ORF13	Antibiotic biosynthesis monooxygenase	116	Antibiotic biosynthesis monooxygenase: *Bacillus cellulosilyticus* DSM 2522 (YP_004095752)<br>Antibiotic biosynthesis monooxygenase: *Cyanothece* sp. PCC 7425 (YP_002484368)	29<br>35
ORF14	Hypothetical protein (truncated)	49	No similarity	–
ORF15	Hypothetical protein (truncated)	44	No similarity	–
ORF16	Hypothetical protein (truncated)	53	No similarity	–
ORF17	Hypothetical protein (truncated)	47	No similarity	–
ORF18	Hypothetical protein (truncated)	66	No similarity	–
ORF19	Alkene monooxygenase β-subunit (AmoA)	342	AmoA: *Rhodococcus rhodochrous* B-276 (BAA07112)<br>Putative propene monooxygenase beta subunit: *Mycobacterium chubuense* NBB4 (ACZ56350)<br>PmoA: *Mycobacterium* sp. M156 (AAS19482)	43<br>41<br>41
ORF20	Alkene monooxygenase coupling/effecter protein (AmoB)	100	AmoB: *Rhodococcus rhodochrous* B-276 (BAA07113)<br>Putative propene monooxygenase coupling protein: *Mycobacterium chubuense* NBB4 (ACZ56351)<br>PmoB: *Mycobacterium* sp. M156 (AAS19483)	60<br>58<br>60
ORF21	Alkene monooxygenase α-subunit (AmoC)	505	AmoC: *Rhodococcus rhodochrous* B-276 (BAA07114)<br>Putative propene monooxygenase alpha subunit: *Mycobacterium chubuense* NBB4 (ACZ56352)<br>PmoC: *Mycobacterium* sp. M156 (AAS19484)	68<br>67<br>66
ORF22	Alkene monooxygenase reductase (AmoD)	344	AmoD: *Rhodococcus rhodochrous* B-276 (BAA07115)<br>Putative propene monooxygenase reductase: *Mycobacterium chubuense* NBB4 (ACZ56353)<br>PmoD: *Mycobacterium* sp. M156 (AAS19485)	56<br>50<br>51
ORF23	Outer membrane protein W	253	OmpW family protein: *Hahella chejuensis* KCTC 2396 (YP_434757)<br>OmpW family protein: *Vibrio splendidus* LGP32 (YP_002395839)	48<br>46
ORF24	iSSod13, transposase<br>integrase catalytic subunit	273	iSSod13, transposase: *Pseudoalteromonas haloplanktis* ANT/505 (ZP_08411520)<br>integrase catalytic subunit: *Shewanella* sp. MR-7 (YP_737813)	85<br>83
